# Changes in the Content of Brain Biogenic Amine Associated with Early Colony Establishment in the Queen of the Ant, *Formica japonica*

**DOI:** 10.1371/journal.pone.0043377

**Published:** 2012-08-15

**Authors:** Hitoshi Aonuma, Takayuki Watanabe

**Affiliations:** Research Institute for Electronic Science, Hokkaido University, Sapporo, Hokkaido, Japan; University of Arizona, United States of America

## Abstract

We examined changes in the content of biogenic amines in the brains of ant queen associated with early colony establishment. In ants, including *Formica japonica*, winged virgin queens lose their wings following copulation, and then start establishing a colony. Significant changes in brain biogenic amine content in the queen are associated with transition from winged virgin queen to wingless mated queen. The levels of serotonin (5HT), octopamine (OA) and dopamine (DA) decreased significantly in the brain of the queen after starting a colony. On the other hand, tyramine (TA) increased significantly in the brain following colony establishment. Catabolized substances of the biogenic amines in the brain were also measured. The levels of N-acetyloctopamine (Nac-OA) and N-acetyltyramine (Nac-TA) in the brain did not show a significant change after the queen established a colony. However, the levels of N-acetylserotonin (Nac-5HT) in the brain were significantly higher in wingless mated queens than in winged virgin queens, whereas levels of N-acetyldopamine (Nac-DA) in the brain were significantly lower in wingless mated queens than winged virgin queens. These results suggest that serotonergic and octopaminergic systems in the brain of the queen change when the mated queen starts to establish a new colony.

## Introduction

The Japanese wood ant, *Formica japonica* is one of the most common ants in Japan. The colonies of this species are largely polygynous and contain thousands of workers and broods [1], [2]. Polygyny (i.e. the presence of more than one queen in a colony of social insects) is thought to be rather common in *Formica* ants [3]. There are hundreds of newborn queens and males living together in a single colony of *F. japonica* in the early summer. When newborn queens of *F. japonica* become sexually mature, they disperse from the nest and perform a nuptial flight. After mating, queens land, shed their wings using their legs, and then start finding places to establish new colonies.

Mating is one of the most important events for animals in their life, and it induces a number of physiological and behavioral changes. It is demonstrated that mating induces behavioral change in butterflies. In the butterfly, *Pieris rapae,* a virgin female accepts and mates with a courting male, but mated females refuse males by displaying a “mate refusal posture”. Biogenic amine systems in particular the serotonergic (5HTergic) system are involved in this switch in female behavior [4]. Similarly, mating has been shown to induce female physiological changes in the brain that alter olfactory coding and preference in the moth, *Spodoptera littoralis* [5]. This suggests to us that mating likely induces physiological changes in *F. japonica* queens. In ants, mated queens start egg-laying earlier and live considerably longer than virgin queens. Additionally, only queens that receive viable sperm from fertile males show increased fecundity [6]. However, it is unclear as to how mated queens switch their behavior and how they modulate physiological states in the brain. Neuromodulation in the brain by biogenic amines is one of the candidates that can change physiological states, which switch behavior after mating.

In nervous systems, biogenic amines are believed to function as neurotransmitters, neuromodulators and neurohormones, and are known to play principal roles in a number of insect behaviors [7]–[9]. The roles of biogenic amines in social behavior and colony organizations have been investigated in insects [10]–[20]. Male crickets increase their aggressiveness towards other males, namely guarding behavior, after copulation [21]. At the same time changes in the level of biogenic amines in the brain and thoracic ganglia have been associated with copulation [22]. Octopaminergic (OAergic) and 5HTergic systems are closely linked to aggressive behavior [23]–[27] and feeding behavior [28]–[30]. In addition, the OAergic system is thought to be linked to nest-mate recognition in social insects such as honeybees [31] and ants [32]. In honey bee, OAergic neuromodulation regulates development of foraging behavior [11], [33] Although the roles of biogenic amines in worker castes of social insects has been studied, little is known about how changes in the contents of brain biogenic amines is associated with early colony establishment in queens.

*F. japonica* is an ideal model system for studying biogenic amines in behavioral state changes. Here we examined the changes in brain biogenic amines content in queen ants and it association with early colony establishment. The amounts of biogenic amines and their catabolized substances in the brain of winged virgin queens and of wingless mated queens were measured by using high-performance liquid chromatography (HPLC) with an electrochemical detection (ECD) system. This work is the first step to elucidate aminergic control associated with early colony establishment in the queen ants.

## Results

**Figure 1:**
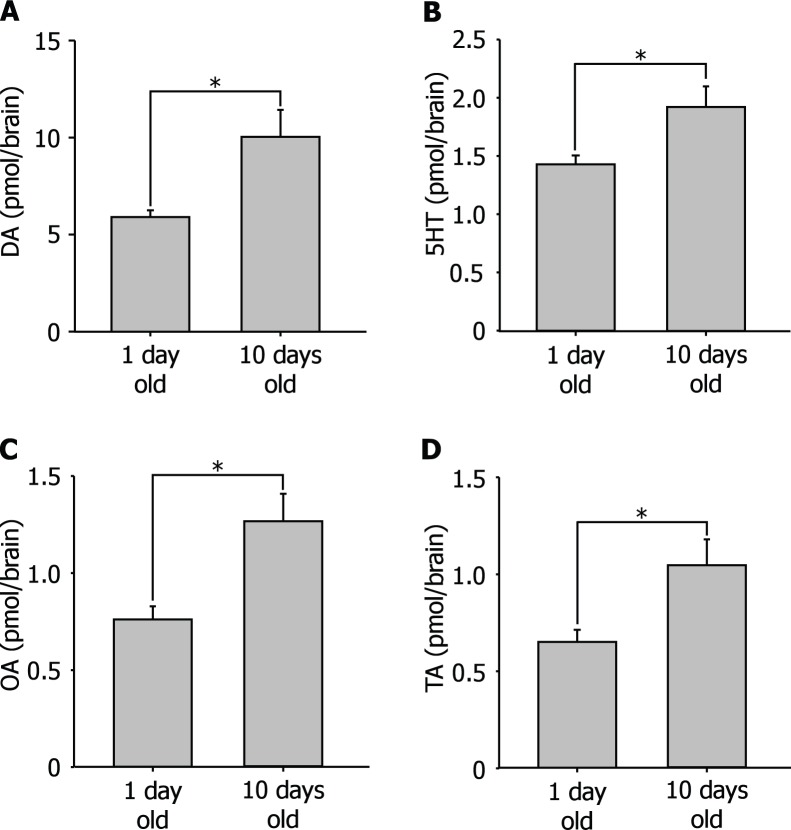
Brain biogenic amines in the newly emerged queens (1 day old) and aged virgin queen (10 days old). A: DA levels in the brain. Brain DA levels in the aged virgin queens were significantly higher than that in the newly emerged queens. B: OA levels in the brain. Brain OA levels in aged virgin queens were significantly higher than that in the newly emerged queens. C: TA levels in the brain. Brain TA levels in the aged virgin queens were significantly higher than that in the newly emerged queens. D: 5HT levels in the brain. Brain 5HT levels were significantly higher than that in the newly emerged queens. (*P<0.05, Student’s *t*-test).

We examined changes in biogenic amine levels of queen brain’s that are associated with the transition from winged virgin queen to wingless mated queen (Table 1). Brain biogenic amine catabolites were also measured in order to estimate the dynamics of biogenic amine changes in the brain. Prior to examining the effect of the mating on the contents of biogenic amines in the brain of queen ants, newly emerged (1 day old) and 10 day old virgin queens were compared (Fig. 1). Some newly emerged queens were kept isolated overnight until the color of the cuticle became black or were kept isolated for 10 days, and then brain amines were measured using HPLC-ECD. The age dependent change in the levels of brain amines was observed in the brain. Levels of the major brain amines in the newly emerged queen were significantly lower than those in 10 day old virgin queens. The amount of DA in the brains of newly emerged queens was 5.90±0.38 pmol/brain (mean±SEM, N = 19). The brain DA level in 10 day old virgin queens increased to 10.1±1.33 pmol/brain (N = 19). The amount of 5HT in the brains of newly emerged queens was 1.45±0.07 pmol/brain (N = 19). The amount of brain 5HT level in 10 day old virgin queen increased to 1.92±0.18 pmol/brain (mean±SEM, N = 19). The amount of OA in the brains of newly emerged queens was 0.76±0.06 pmol/brain (N = 19). The amount of brain 5HT level in 10 day old virgin queen increased to 1.26±0.14 pmol/brain (N = 19). The amount of TA in the brains of newly emerged queens was 0.65±0.06 pmol/brain (N = 19). The amount of brain TA level in 10 day old virgin queen increased to 1.05±0.13 pmol/brain (N = 19).

**Table 1:** Contents of biogenic amines and their N-acetylated substance in the ant brain.

	Virgin queen	Mated queen	Worker
	(N = 10)	(N = 10)	(N = 10)
DA	4.73±0.22	2.43±0.20*	5.02±0.31
Nac-DA	1.69±0.29	1.14±0.10*	1.55±0.21
TA	0.41±0.04	1.17±0.22*	0.45±0.05
Nac-TA	1.10±0.11	1.03±0.11	0.52±0.02
OA	1.50±0.20	0.40±0.03*	1.34±0.10
Nac-OA	1.00±0.17	1.01±0.12	0.18±0.03
5HT	1.71±0.13	1.39±0.07*	1.89±0.07
Nac-5HT	0.21±0.02	1.12±0.14*	0.28±0.04

The levels of brain biogenic amines were compared between virgin and mated queens and tested using Student’s t-test (*P<0.05). As a reference, the contents of biogenic amine levels in foraging workers that interact with nest mates were measured [67].

(Mean ± SEM, pmol/brain).

**Figure 2:**
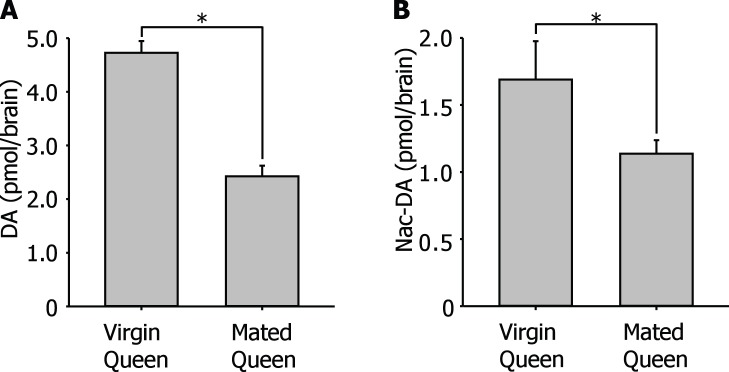
Change in levels of DA and its related substances in the brain of queen associated with early colony establishment. A: Change in level of DA in the brain of queen associated with early colony establishment. The level of DA in the brain of virgin queen was around 5 pmol/brain. The level of DA in the brain decreased significantly after a queen starts establishing a colony. B: The level of Nac-DA in the brain of queen. The level of Nac-DA in the brain significantly decreased after a queen starts establishing own colony. (*P<0.05, Student’s *t*-test).

The DA levels in the brain of the ants are maintained by ongoing social interactions [19]. DA is generated from L-tyrosine through L-DOPA by the enzymes tyrosine hydroxylase and aromatic L-amino acid decarboxylase, respectively. In our HPLC experiment we failed to measure both tyrosine and DOPA in the brain because the peaks of these two substances on the chromatogram appear as front peaks. DA and N-acetyldopamine (Nac-DA) were detected in the brain of both winged virgin queens and wingless mated queens (Fig. 2A, B). The brain DA levels of winged virgin queens were significantly higher than those detected in wingless mated queens (Fig. 2A). The amount of DA in the brains of winged virgin queens was 4.72±0.22 pmol/brain (N = 10). This value was similar to the levels of DA found in the brains of workers (5.02±0.31 pmol/brain, N = 10). On the other hand, the amount of DA in the brains of wingless mated queens (2.45±0.20 pmol/brain, N = 10) was significantly less than in virgin queens or workers. The amount of Nac-DA in the brains of winged virgin queens (1.69±0.29 pmol/brain, N = 10) was significantly less than in wingless mated queens (1.14±0.10 pmol/brain, N = 10; Fig. 2B).

**Figure 3:**
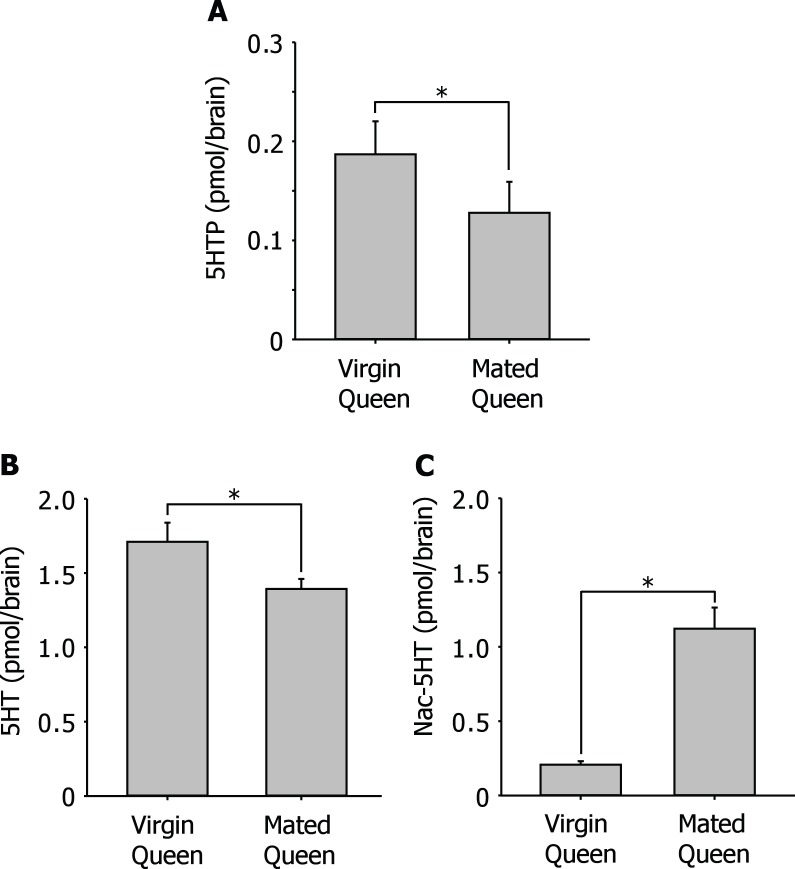
Change in levels of OA and its related substances in the brain of queen associated with early colony establishment. A: Change in level of TA in the brain of queen associated with early colony establishment. The content of TA in the brain was less than 0.5 pmol/brain. The level of TA in the brain increased significantly after a queen starts establishing own colony. B: The level of Nac-TA in the brain of queen. There was no significant change in the level of Nac-TA between winged virgin queen and wingless mated queen. C: Change in level of OA in the brain of queen associated with early colony establishment. The content of OA in the brain of virgin queen was about 1.5 pmol/brain. The level of OA in the brain decreased significantly after a queen starts establishing own colony. E: The level of Nac-OA in the brain of a queen. No significant change in the level of Nac-OA in the brain was observed between winged virgin queen and wingless mated queen. (*P<0.05, Student’s *t*-test).

OA is involved in social behavior and links to nest-mate recognition in ants [32]. OA is generated from L-tyrosine but through different synthetic pathways than DA generation. The levels of TA and OA in the brains of winged virgin queens were similar to those in the brains of workers (Table 1). However the TA levels of the brains of winged virgin queens were significantly less than those of wingless mated queens (Fig. 3A). The levels of TA in the brains of winged virgin queens were 0.41±0.04 pmol/brain (N = 10) and were significantly increased to 1.17±0.22 pmol/brain (N = 10) after the queens start to establish a new colony. On the other hand, there was no significant difference between the levels of Nac-TA in the brains of winged virgin queens (1.10±0.11 pmol/brain, N = 10) and those of wingless mated queens (1.03±0.11 pmol/brain, N = 10) (Fig. 3B).

The amount of OA in the brain of winged virgin queens was 1.50±0.20 pmol/brain. The content of OA in the brain of wingless mated queens (0.40±0.03 pmol/brain) was significantly less than in winged virgin queen (Fig. 3C). On the other hand, the amount of catabolized substance Nac-OA did not change after establishment of a colony (Fig. 3D). The amount of Nac-OA in the brain of winged virgin queens was 1.00±0.17 pmol/brain and wingless mated queen was 1.01±0.12 pmol/brain.

**Figure 4:**
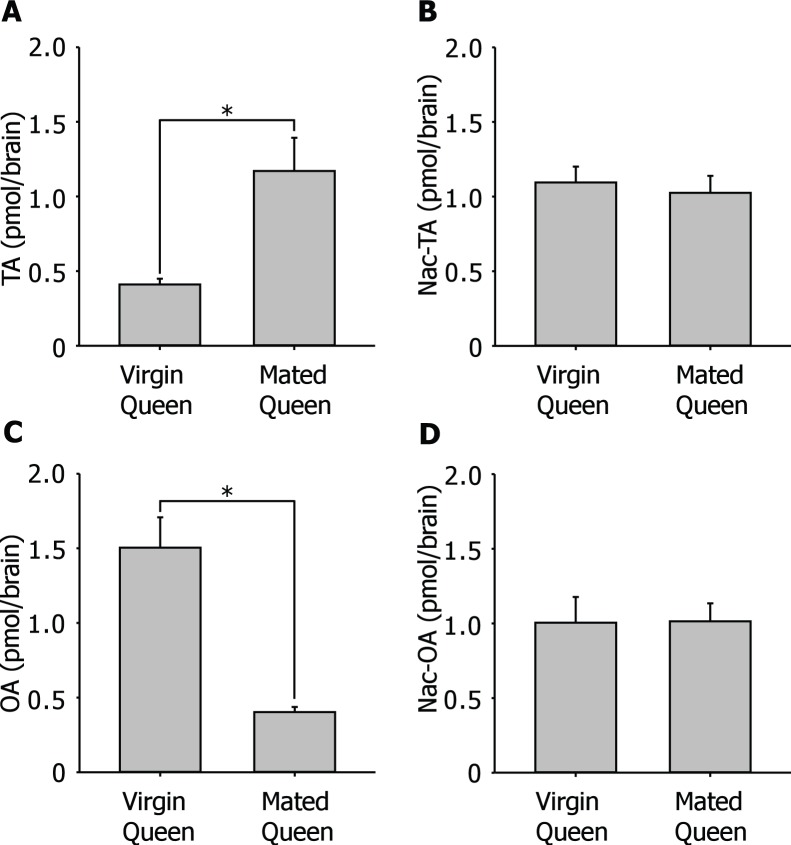
Change in levels of 5HT and its related substances in the brain of queen associated with early colony establishment. A: Change in level of 5HTP in the brain of queen associated with early colony establishment. 5HTP significantly decreased after queen started establishing a colony. B: Change in level of 5HT in the brain of queen associated with early colony establishment. 5HT significantly decreased after queen started establishing a colony. C: Change in level of Nac-5HT in the brain of queen associated with early colony establishment. Nac-5HT significantly increased after queen started establishing a colony. (*P<0.05, Student’s *t*-test).

5HT in the brain is also thought to link to aggressive behavior in ants [23]. The levels of 5HTP that is a precursor of 5HT in the brains of wingless mated queens were significantly less than those of winged virgin queens (Fig. 4A). The levels of 5HT in the brains of winged virgin queens were 1.71±0.13 pmol/brain (N = 10). This value was similar to the levels of 5HT in the brains of workers. On the other hand, the levels of 5HT in the brains of wingless mated queens (1.39±0.07 pmol/brain, N = 10) were significantly less than those of winged virgin queens (Fig. 4B). The balance between generated and catabolized 5HT determines the functional level of 5HT. Therefore the levels of Nac-5HT in the brains were also examined. The levels of Nac-5HT in the brains of winged virgin queens (0.21±0.02 pmol/brain, N = 10) were significantly less than those in wingless mated queens (1.12±0.14 pmol/brain, (N = 10) (Fig. 4C).

## Discussion

Biogenic amines in ants have been demonstrated to play important roles in initiating social behavior [14], [19], [32], [34], [35]. The changes in the content of the biogenic amines in the brain were examined in both winged virgins and wingless mated queens of *F. japonica* in this study. Significant changes in the contents of brain biogenic amines (DA, OA and 5HT) in the queens were associated with the transition from virgin to mated queen.

The DA levels in the brains were significantly lower in mated queens than in virgin queens. The levels of brain Nac-DA, a catabolite of DA, were also lower in the mated queens than in the virgin queens. These results suggest that the DAergic system in the brain is suppressed when the mated queens start establishing new colonies. It is widely known that different environmental stimuli, such as temperature, mechanical or chemical stimuli, population density, starvation and social interaction, modulate biogenic amine levels in the insect brain [19], [36]–[41]. We can thus add another ‘stimulus’ that alters DA levels in the brain - the transition from a winged to wingless queen as a result of mating.

The virgin queens stay in their parental nest until they become sexually mature. They are fed by workers through trophallaxis and can receive grooming from them. After copulation, a mated queen seeks out a new location in order to establish a new colony. The energetic demands during the establishment phase are high, as the queen needs to nurse her brood without eating anything until her workers grow up to become foragers. Social isolation with starvation stress for 10 days increased brain DA levels in the virgin queens in this study. On the other hand, starvation stress decreases DA levels in the brains of the worker ants *F. japonica*, and the decreased DA levels in the brain caused by starvation stress are restored by social interaction with nest-mates [19]. Further study is necessary to understand the roles of brain DA in different castes.

Among the three biogenic amines examined in this study, DA is related with reproductive behavior in insects. Increase in brain DA levels in the eusocial wasps is linked to egg-laying behavior [42]. DA also relates with copulation behavior in female *Drosophila* [43], and DA-depleted females are significantly less receptive to males. In social insect such as ants, bees and wasps, pheromones produced by queens are widely believed to affect worker reproduction. In a queenless colony of honeybees, the worker brain DA levels are increased which results in ovarian development [44]. Mandibular gland pheromone of the queen honeybee has been demonstrated to affect gene expression in the brain [45] as well as modulate action of brain DA [46], [47] in worker bees. This study is the first step in revealing how biogenic amines change in the brain of queens when queens start establishing a colony after mating. As a next step, we will need to examine the change in the levels of brain biogenic amines associated with the colony development to elucidate whether mating itself changes the contents of biogenic amines in the brain or whether social interaction regulates biogenic amine systems.

The OAergic and 5HTergic systems in the brain are linked with initiating many kinds of behaviors in insects. For example, the OAergic and 5HTergic systems as well as the DAergic system in the brain are involved in learning and memory in insects [48]–[50]. It has also been demonstrated in silkmoths that OA and 5HT increase sensitivity of individuals to pheromones [51]–[53]. The OAergic system in the brain is also linked to nest-mate recognition in social insects such as honeybees [54] and ants [32]. In the fire ant, queenless workers have lower levels of brain OA, which is associated with a reduced ability to recognize nest mate [32]. In addition, OA is thought to be involved in initiating social behavior. Social isolation modulates trophallaxis in the ants *Camponotus fellah* [55] and decrease brain OA in *F. japonica* [19]. The application of OA changes the duration of trophallaxis in socially deprived workers *C. fellah* [34], [35]. Worker honeybees change their jobs, as they grow older. Younger workers perform nursing while the oldest workers in the colony forage for foods outside the hive. Division of labor in the colony is controlled by the interaction between the needs of the colony and the physiological states of the nest mate [56]–[58]. OA influences division of labor in honeybee, and increase in brain OA levels are associated with increases in foraging behavior [13]. OA treatments cause an increase in responsiveness to brood pheromone and a decrease in responsiveness to social inhibition in honeybee [15]. In ants, age-related changes in the contents of biogenic amines have also been reported. The levels of brain 5HT and DA increase with age in the minor ant *Pheidole dentata*, however OA levels did not change with age in the ant [17]. In ants, minor workers increase repertory of task performance [59]. The number of 5HTergic neurons at optic lobes increase with age in *P. dentata* [60]. These findings suggest to us that physiological change in the brain and behavior with age are modulated by 5HTergic system in the brain in ants. On the other hand, our experiments demonstrated that the levels of brain DA, OA, TA and 5HT increased with aging to become sexually matured in virgin queens, and that the levels of brain 5HT, OA and DA decreased after mating. In our experiments, queens started egg-laying soon after copulation, and the age difference between virgin queens and mated queens were about 1 week in this study. Considering life span of queens, it is unlikely this age difference affected the change in the contents of brain biogenic amines, and suggests that the results were solely a result of mating.

Here we demonstrated that the levels of brain OA were significantly lower in mated queens than in virgin queens. TA and OA are decarboxylated substances of the amino acid L-tyrosine and TA is the biological precursor of OA. The levels of brain TA were significantly higher in mated queens than in virgin queens, while the levels of brain OA were significantly lower in mated queens than in virgin queens. Our data suggest to us that TA itself functions as a neuromodulator in the brain and is related with colony establishment. In insect, TA and OA function as independent neurotransmitters, acting through specific G-protein coupled receptors [61]. On the other hand, we found that the levels of Nac-TA and Nac-OA were not different between virgin and mated queens. This suggests to us that the OAergic system of the queen is suppressed by decreased biosynthesis of OA in the brain when the queen starts to establish a colony. In the worker *F. japonica* ants, the level of OA in the brain was almost similar to that in the brain of virgin queens (Table 1), although the size of the worker brain is slightly smaller than that of the queen brains (personal observation). However, the levels of Nac-OA in the brain of the queen were much higher than that of the worker. This suggests that the OAergic system in the brain of the queens is suppressed compared to that of the workers. In addition to the drastic changes in the OAergic system, the levels of 5HT in the brain were also significantly lower in mated queens than in virgin queens. The levels of brain 5HTP, the biological precursor of 5HT, were significantly lower in mated queens than in virgin queens, but the levels of 5HT catabolite, Nac-5HT, were significantly higher in mated queen than in virgin queens. These data indicate that the 5HTergic system in the brain of the queens is also suppressed when they start establishing new colonies.

Biogenic amines in the brain are linked with the endocrine system of insects [62]. In social insects, the endocrine system has been believed to be one of the important mechanisms for the control of social behavior. Juvenile hormone (JH) and ecdysteroids are the key molecules to determine caste and division of labor in social insects (ant: [63], honeybee: [54]). JH and ecdysteroids function as primary regulators of behavior and ovarian activity in the ants [64]. JH is involved in regulating reproductive maturation in the queen and behavioral maturation of worker tasks in the social wasps, [65]. The production of JH is regulated by the biogenic amine system in insect: in honeybee, OA stimulates JH production in the corpora allata (CA) [66], and in cricket, OA suppresses JH III production in the CA *in vitro* [62]. Further investigation is necessary to understand how biogenic amines in the brain affect the endocrine system in the queen ants and how biogenic amine systems in the brain modulate behaviors of queens. In this study we gain new insight into the physiological state changes in the brain of queen ants associated with early colony establishment.

## Materials and Methods

### Animals

Colonies of ants *Formica japonica* were collected at Hokkaido University in June 2011 and 2012. They were installed in artificial plaster nests and reared at 26°C under L/D 12∶12 (lights on at 6∶00 h). The colonies contained many cocoons of males and queens and several hundreds of workers. Ants were fed a diet of insect jelly (Marukan Co., Ltd, Osaka, Japan), dead crickets and water *ad libitum*. Virgin *F. japonica* queens remain for several days in the colony until they reach sexual maturity. They then, together with male ants, exit the colony and perform a nuptial flight, typically in the early summer. Queens that have wings were collected in the artificial colony and they were expected to be sexually immature virgin. Queens of *F. japonica* were collected with male ants at the outside of the colony and kept together several days. After copulation, winged queens were kept individually in a small plaster box (4 cm×5 cm×1.5 cm) as an artificial nest for establishing their colony. Mated queens started laying eggs within 1 or 2 day(s) after mating, and nursing their brood. Then we used wingless queens for our experiments after we confirmed they started nursing eggs and brood (early colony establishment stage). In order to examine the effects of aging, some virgin queens were kept isolated for 10 days before brain biogenic amines measurement as a control.

### Measurement of Brain Biogenic Amines

A single ant was quickly frozen using liquid N_2_. The brain of a frozen ant was dissected out in the ice-cold ant saline (128.3 mM NaCl, 4.7 mM KCl, 1.63 mM CaCl_2_, 6 mM NaHCO_3_, 0.32 mM NaH_2_PO_4,_ 82.8 mM trehalose, pH 7.4). A single brain of a queen was collected into a micro glass homogenizer and homogenized in 50 µl of ice-cold 0.1 M perchloric acid containing 5 ng of 3, 4-dihydroxybenzylamine (DHBA, SIGMA, St Louis, MO, USA) as an internal standard. After centrifugation of the homogenate (0°C, 15000 rpm, 30 min), 40 µl of supernatant was collected. Biogenic amine in the brain was measured using high-performance liquid chromatography (HPLC) with electrochemical detection (ECD). The HPLC-ECD system was composed of a pump (EP-300, EICOM Co., Kyoto, Japan), an auto-sample injector (M-504, EICOM Co., Kyoto, Japan) and a C18 reversed-phase column (250 mm×4.6 mm internal diameter, 5 µm average particle size, CAPCELL PAK C18MG, Shiseido, Tokyo, Japan) heated to 30°C in the column oven. A glass carbon electrode (WE-GC, EICOM Co.) was used for electrochemical detection (ECD-100, EICOM Co.). The detector potential was set at 890 mV versus an Ag/AgCl reference electrode, which was also maintained at 30°C in a column oven. The mobile phase containing 0.18 M chloroacetic acid and 16 µM disodium EDTA was adjusted to pH 3.6 with NaOH. Sodium-1-octanesulfonate at 1.85 mM as an ion-pair reagent and CH_3_CN at 8.40% (v/v) as an organic modifier were added into the mobile phase solution. The flow rate was kept at 0.7 ml/min. The chromatographs were acquired using the computer program PowerChrom (eDAQ Pty Ltd, Denistone East, NSW, Australia). The supernatants of samples were injected directly onto the HPLC column. After the acquisition, they were processed to obtain the level of biogenic amines in the same sample by the ratio of the peak area of substances to the internal standard DHBA. We used a standard mixture for quantitative determination that contained amines, precursors and metabolites. Twenty compounds at 100 ng/ml each were DL-3, 4-Dihydroxy mandelic acid (DOMA), L-β-3,4-Dihydroxyphenylalanine (DOPA), L-Tyrosin (Tyr), N-acetyloctopamine (Nac-OA), (−)-noradrenaline (NA), 5-Hydroxy-L-tryptophan (5HTP), (−)-adrenaline (A), DL-Octopamine (OA), 3,4-Dihydroxybenzylamine (DHBA, as an internal standard), 3,4-Dihydroxy phenylacetic acid (DOPAC), N-acetyldopamine (Nac-DA), 3,4-Dihydroxyphenethylamine (DA), 5-Hydroxyindole-3-acetic acid (5HIAA), N-acetyltyramine (Nac-TA), N-Acetyl-5-hydroxytryptamine (Nac-5HT), Tyramine (TA), L-Tryptophan (Trp), 3-Methoxytyramine (3MTA), 5-Hydroxytryptamine (5HT), 6-Hydroxymelatonin (6HM). Nac-OA Nac-DA and Nac-TA were synthesized by Dr. Matsuo (Keio University, Japan). All other substances were purchased from SIGMA.

Differences in the levels of biogenic amines were tested using Student’s *t*-test (*P*<0.05).
